# Structural Insights into RbmA, a Biofilm Scaffolding Protein of V. Cholerae

**DOI:** 10.1371/journal.pone.0082458

**Published:** 2013-12-05

**Authors:** Manuel Maestre-Reyna, Wen-Jin Wu, Andrew H.-J. Wang

**Affiliations:** Institute of Biological Chemistry, Academia Sinica, Taipei, Taiwan; Universitätsklinikum Hamburg-Eppendorf, Germany

## Abstract

*V. cholerae* can form sessile biofilms associated with abiotic surfaces, cyanobacteria, zoo-plankton, mollusks, or crustaceans. Along with the vibrio polysaccharide, secreted proteins of the rbm gene cluster are key to the biofilm ultrastructure. Here we provide a thorough structural characterization of RbmA, a protein involved in mediating cell-cell and cell-biofilm contacts. We correlate our structural findings with initial ligand specificity screening results, NMR protein-ligand interaction analysis, and complement our results with a full biocomputational study.

## Introduction

In estuarine and brackish waters[1], *V. cholerae* may swim freely, or grow in sessile biofilms associated with abiotic surfaces, zoo-plankton, mollusks, or crustaceans[2]. *Vibrio cholerae* biofilms are involved in many aspects of the pathogen's life-cycle[3,4], as well as constituting a possible source of antibiotic resistances[5]. Along with the vibrio polysaccharide (VPS)[6], secreted proteins of the rbm gene cluster, including RbmA, are key to biofilm ultrastructure[7]. 

RbmA is a 26.4 KDa protein, with putative carbohydrate binding activity[8], which is found within the biofilm matrix, mediating cell-cell and cell-biofilm contacts[9]. Even though RbmA is not essential for biofilm biogenesis, it confers a high degree of mechanical stability to *V. cholerae* sessile communities by a mechanism which is not well understood. Here we present the RbmA crystal structure, both in its apo form and complexed with an artificial ligand. We have also performed ligand binding screening; and the results were confirmed via saturation-transfer difference (STD) NMR experiments[10]. We then proceeded to define ligand binding mode *in-silico*. We found RbmA to be a two-domain protein, functionally arranged as a dimer, with a high specificity for sialic acid, rhamnose, fucose, galactose and N-acetyl galactosamine. We propose a model for RbmA mediated biofilm-cell cross-linking, based on our structural and functional studies. This model helps advance our understanding of matrix scaffolding in *Vibrio* biofilms, and the study of the mechanisms by which bacteria associate themselves into communities. Our proposed model may serve as the basis for a wide variety of *in vivo* studies, correlating the molecular with the ultrastructural levels in biofilm architecture. Furthermore, the determination of RbmA specificity is a first step toward the development of scaffolding inhibitors.

## Methods

### Cloning, production and purification of RbmA

An *E. coli* optimized synthetic RbmA gene based on the sequence from *V. cholerae* O1 (geneID 7855157) was designed, omitting the N-terminal secretion signals, and adding NdeI and XhoI restriction sites for cloning purposes. The secretion signal was predicted using SignalP[11]. The gene was then cloned into the pET28a vector, which was used to transform chemo-competent *E. coli* BL21(DE3) cells. Protein production was carried out in LB medium, via IPTG induction (1 mM final concentration), at 37 °C, and 160 rpm.

Alternatively, Se-Met derived protein destined for single wavelength anomalous diffraction (SAD) phasing was produced using the Overnight Express Autoinduction system 2 (Novagen), as described in the handbook.

*E. coli* cells carrying RbmA were then harvested, resuspended in loading buffer (20 mM Tris/HCl pH 8, 100 mM NaCl, 5 mM imidazol) complemented with EDTA free protease inhibitor cocktail (Roche), and lysed using a cell disruptor (Constant Systems LTD). Cell debris was removed via centrifugation, and supernatant was filtered and loaded on a 20 mL Ni Sepharose 6 Fast Flow column (GE). After loading, and washing, the protein was eluted via a linear gradient with elution buffer (loading buffer + 500 mM imidazol). The protein was concentrated using Amicon concentrators, and loaded into a Superose 6 size exclusion chromatography column, pre-equilibrated in crystallization buffer (20 mM Tris/HCl pH, 100 mM NaCl). Main peak fractions were collected and re-concentrated to around 25 mg/mL, and stored at 4 °C.

### High-throughput glycan array binding assays

The glycan array used by the Consortium for Functional Glycomics (CFG) consists of different groups of oligosaccharides that are presented by mammalian cells. RbmA was fluorescently labeled using an AlexaFluor 488 SPD kit (Invitrogen) and applied to CFG array V5.1 chips at 200 μg/mL. Alternatively, RbmA was directly applied to the glycan array, with binding activity being detected via fluorescent anti-his-tag antibodies. Chip surfaces where repeatedly washed and remaining fluorescence was measured and quantified.

Each binding event was repeated six times, with the highest and lowest value discarded. The remaining data were averaged, and standard deviations were calculated.

#### Samples for NMR measurements

The NMR samples contained 20-fold molar excess of sugars added to RbmA (0.1 mM dimer concentration) in pH 7.4 buffers containing 20 mM potassium phosphate, 100 mM NaCl, 8% D_2_O (for locking purpose) and 0.01 mM 4,4-dimethyl-4-silapentane-1-sulfonic acid (DSS, for chemical shift referencing). The pH values for both sugars and RbmA were adjusted to the same value prior to mixing (less than a 0.05 pH unit differences, if any).

#### Saturation transfer difference (STD) experiments [10]

For the on-resonance irradiation experiment, a train of 50 msec Gaussian shape pulses were applied to the protein signals at -0.37 ppm (up field shifted methyl groups) for two seconds, one second relaxation delay was applied. For the off-resonance irradiation experiment, the same selective pulse was applied to -20 ppm where no signals occurred. A 65 msec spin lock period was employed to filter out protein resonances. Water suppression was achieved using 3919 WATERGATE scheme [12]. 7424-9600 scans were accumulated. The data were collected at 298 K on a Bruker 800 MHz spectrometer equipped with a CryoProbe. 

### Crystallization of RbmA

RbmA was subjected to an initial sparse matrix screening, followed by optimization of promising hits. Native, and selenomethionine derived RbmA crystals were grown in conditions containing 0.1 M Bis-tris pH 5.5 – 6.5, and 1.5 - 2.5 M NaCl. The best diffracting native RbmA crystal (4be6) was grown in the presence of 10 mM sialic acid. However, no sialic acid could be detected in the electron density map of the RbmA diffraction data. 18-crown-6 co-crystals were obtained in 0.1M HEPES pH 7 – 7.5, 0.2 M CaCl_2_, 30% PEG400 (V/V). Native RbmA crystals took between 2 - 3 days to grow fully, while 18-crown-6 co-crystals were visible almost immediately after pipetting, and grew larger within two days.

### Data collection, phase solution, and refinement

Data collection took place at the 13B and C beamlines at the National Synchrotron Radiation Center (Hsinchu, Taiwan), and at the 12B2 beamline Spring-8 (Hyogo, Japan). ApoRbmA crystals and SeMet derivates belonged to space group *P*4_1_2_1_2, and were solved by single anomalous dispersion (SAD) using Shelx[13]. All other crystals, including co-crystals, were solved via molecular replacement using the Phaser[14] software package. Refinement was carried out using Refmac5[15], included in the CCP4 suite[16], and Coot[17]. Secondary structure assignments were performed with STRIDE[18], and figures were rendered using PyMOL[19]. Data collection details and refinement statistics are listed in Table 1.

**Table 1:** Data collection and Refinement details.

	ApoRbmA	SeMet_RbmA	RbmA Crown
**Data collection**			
Space group	P4_1_2_1_2	P4_1_2_1_2	P3_1_
Cell dimensions			
*a*, *b*, *c* (Å)	118.22, 118.22, 104.98	118.89, 118.89, 104.78	136.88, 136.88, 116.31
α, β, γ (°)	90, 90, 90	90,90,90	90,90,120
Wavelength (Å)	1.00000	0.96000	0.97622
Resolution (Å)	2.00 - 24	2.46 - 50	2.6 - 22
	(2.00 - 2.03)	(2.46 - 2.5)	(2.6 - 2.69)
*R*_merge_	6.5 (55.1)	7.2 (37.5)	9.3 (93.1)
I/σI	37.8 (5.2)	45.4 (9.74)	21.6 (2.44)
Completeness (%)	99.7 (100.0)	99.86 (98.97)	100 (100)
Redundancy	13.2 (11.6)	9.8 (9.8)	5.9 (5.8)
**Refinement**			
Resolution (Å)	2.05	2.46	2.6
No. reflections	50815	26340	75002
*R*_work/_ *R*_free_	13.29/16.09	18.38/23.64	22.09/26.02
No. atoms			
Protein	3570	3562	14166
Water	540	307	290
Crown-ethers			144
B-factors			
Protein	25.90	39.26	55.11
Water	41.10	37.02	45.08
Crown-ethers			44.08
R.m.s deviations			
Bond lengths (Å)	0.012	0.010	0.009
Bond angles (°)	1.46	1.41	1.34

Values for highest resolution shell are in parenthesis.

### Docking and molecular dynamics simulations

Suitable binding pockets were first approximated using the InCa-sitefinder server[20]. Intermolecular A/B domain structures were submitted to the server in order to avoid smaller binding pockets being masked by the wide groove (Figure S4 in File S1). Once preliminary binding pockets had been isolated, they were confirmed via docking with sialic acid, using Vina[21]. In order to determine the grid sizes and to visualize ligand poses, the PyMOL[19] Autodock/Vina plugin was employed[22]. Sialic acid was prepared for docking using a glycam06[23] template. Hydrogen atoms were added to the template using the ADT software package, which is capable of protonating heavy atom according to pH, valence, and charge.

MD simulations were carried out using Amber12[24]. For the 60 ns simulations in the presence and absence of the 18-crown-6 ligand, the protein model based on structure 4bei was surrounded with a 20 Å TIP3PBOX water box. For the modeling of the crown ether, the Antechamber software and the General Amber Force Field (GAFF) were employed. The protein was then subjected to energy minimization for 10000 cycles, followed by equilibration with weak restraints for 100 ps. Finally, the system was equilibrated to 300 K and 1 atm, and run for 60 ns. For the docking experiments, protein-glycan complexes were selected using the docking energy criteria (Table S1 in File S1), but also taking into account the binding mode. Hence, two ligand conformations were selected for the D- and O-loop protein conformations, but only one for the wide groove binding pocket. Further processing of the protein-glycan complexes was analogous to the RbmA●18-crown-6 complex, but the water boxes were kept at 10 Å, and simulations were only 20 ns long. Finally, the RbmA●polylactose complex was generated with Vina by joining the two most favored conformations of a polylactose decamere. The resulting model was then treated following guidelines reported previously in literature for the treatment of protein-glycan complexes[25].

Trajectories were evaluated with VMD[26], which was also used for movie rendering. The RMSD data was plotted with Qtiplot[27].

### Multiple alignment of RbmA sequences

The presence of RbmA in the genomes of all sequenced strains presented in reference [28] was first determined via Blastp[29,30]. Once the subset of RbmA^+^ *V. cholerae* strains was identified, a multiple alignment of all RbmA sequences was performed with COBALT[31] and displayed with ClustalX[32]. 

## Results and Discussion

**Figure 1:**
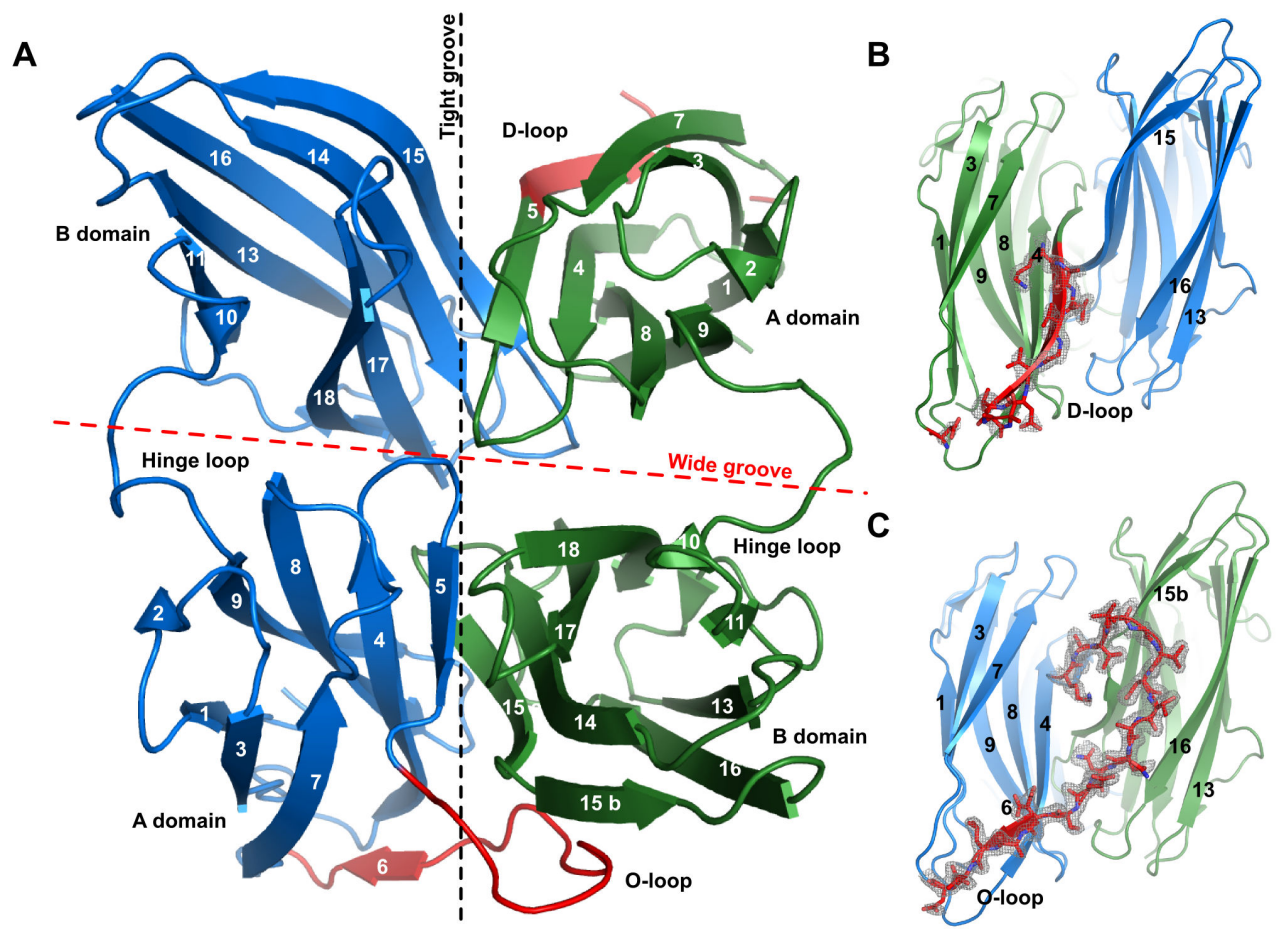
The RbmA structure. A: ApoRbmA general fold. RbmA is a functional dimer (monomers in blue and green). At the tight groove interface (black dotted line), between β-strands five and seven, RbmA can adopt two distinct conformations (red, D- and O-loop). B: Detail of the D-loop conformation, which results in an elongation of β5, the loss of β6 and the disorganization of the loop beyond β5. C: Detail of the O-loop conformation, which presents an elongated loop between β5 and β6. Omit electron at 1σ (0.24 e/Å^3^), carved to 1.6 Å.

The RbmA crystal structure (2.05 Å resolution; PDBID 4be6) revealed an all-beta dimer (Figure 1 A), which was similar to recently published results[33], with the main differences at the dimer interfaces. Each monomer is composed of two structurally similar (1.4 Å RMSD), but distinct (24% sequence identity) domains (A- and B-domains). In the dimer, each A-domain interacts loosely with the B-domain of the same chain, forming a wide groove, and with the B-domain of the other polypeptide, forming a tight groove. Within each chain the A and B domains are connected by a flexible hinge loop (Figure 1). The wide groove builds on electrostatic interactions along charged stripes on the inner surface of each of the domains (Figure S1 in File S1), while the tight groove is firmly held together by interlocking β-sheets (Figure 1 A). What our structure shows that the previously published do not, is that, within this region, strand β5 can show two conformations in the same dimer. In the disordered loop (D-loop) conformation, β5 is continuous, and is followed by a long, flexible loop, which was not visible in the electron density map (Figure 1B). On the other hand, in the ordered loop (O-loop) conformation, β5 is very short, replaced by a well-defined loop, which tightly hugs the B-domain across the tight groove (Figure 1C). The O-loop is followed by the short β6, which is absent in the D-loop conformation. The O-loop partially shields a positively charged patch on the wide groove from solvent, exposing negative charges on the hitherto fully positive surface of the tight groove, and deepening it into a 417 Å^3^ pocket (O-loop pocket) (Figure S2 in File S1).

**Figure 2:**
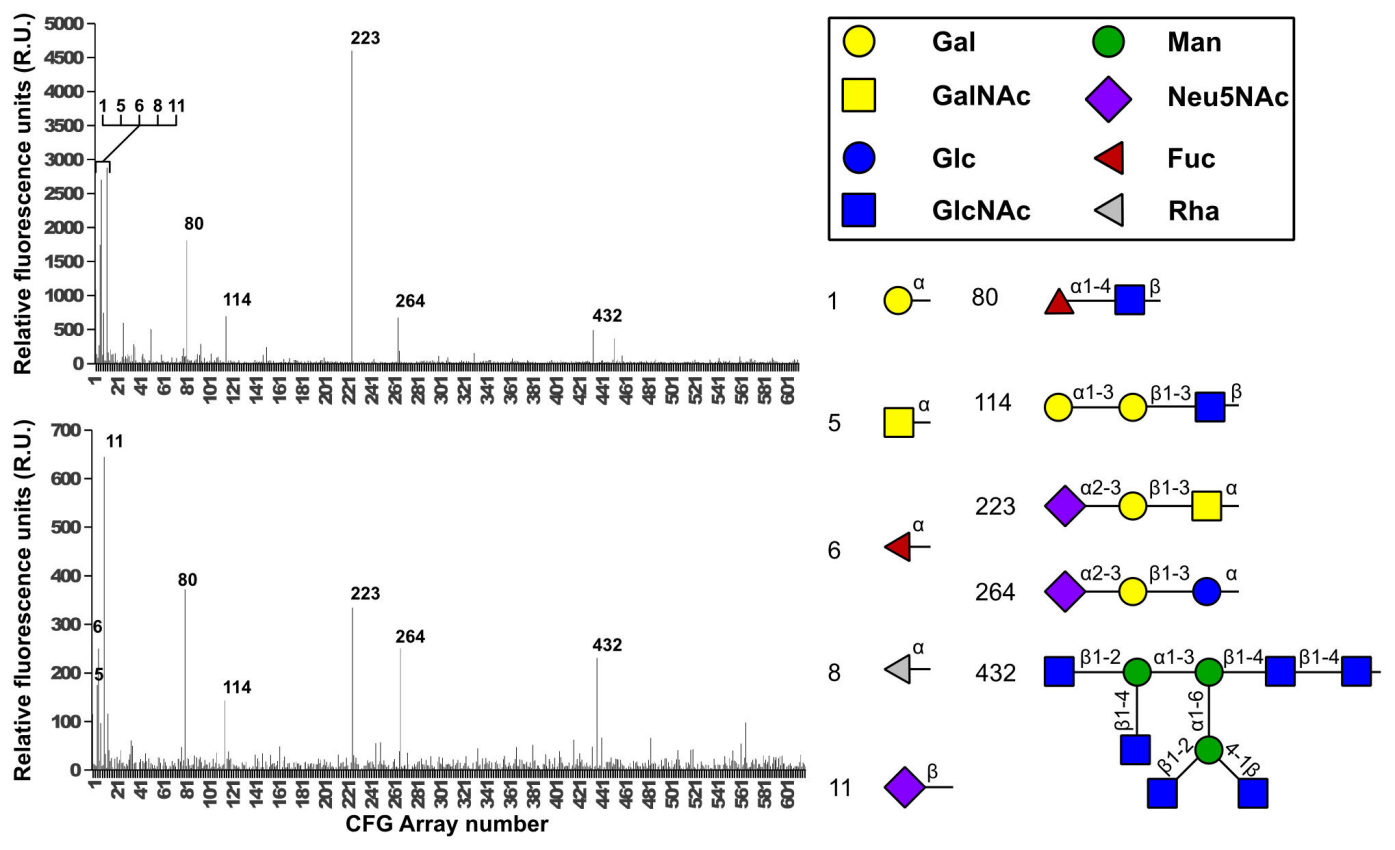
Glycan array assays for RbmA. Top binding glycans are highlighted according to their glycan id, and described in detail on the right side, using the CFG graphic notation. A: RbmA was labeled with a fluorophore and bound to the CFG glycan array V5.1. B: Unlabeled RbmA was detected via anti-his-tag antibody.

In order to assess the putative glycan binding capacity of RbmA, we undertook high-throughput, semiquantitative binding studies with the Consortium for Functional Glycomics (CFG). The AlexaFluor 488 labeled RbmA binding profile provides a detailed specificity overview against a wide variety of glycans (Figure 2). We further confirmed these binding results, and discarded any false positives or negatives produced by the labeling on primary amines, by repeating the experiment with unlabeled RbmA, which was then detected via fluorescent anti-his antibodies (Figure 2). RbmA seemed to prefer simple mono- to trisaccharides. Sialic acid (Neu5NAc) containing glycans 11 and 223 were the top binders, while fucose-containing glycans also figured prominently (glycans 6 and 80). Finally, galactose, rhamnose, and N-acetyl galactosamine (glycans 1, 8, and 5, respectively) were bound at slightly lower strengths than glycan 80. Glycan 5, in fact, bound solely as α-GalNAc-serine, which is the smallest unit for O-glycosilation[34]. RbmA showed a clear preference for α-linked galactose, while sialic acid was preferably bound in the β form, while the same glycans, with the opposite linkages, remained consistently free (glycans 10, and 11 for α-sialic acid, 12 for β-galactose). Such a preference explains the low number of binding partners for RbmA on the glycan array, since these glycans are underrepresented in it. α-rhamnose is the only rhamnose containing sugar in the array, and only 65 glycans out of the 610 contain α-galactose; the presence of β-sialic acid is even lower, with 6 glycans presenting it. Fucose, on the other hand, is not underrepresented, but every fucoside except glycans 6 and 80 contained a β-galactose moiety, which seems to be detrimental to RbmA binding.

**Figure 3:**
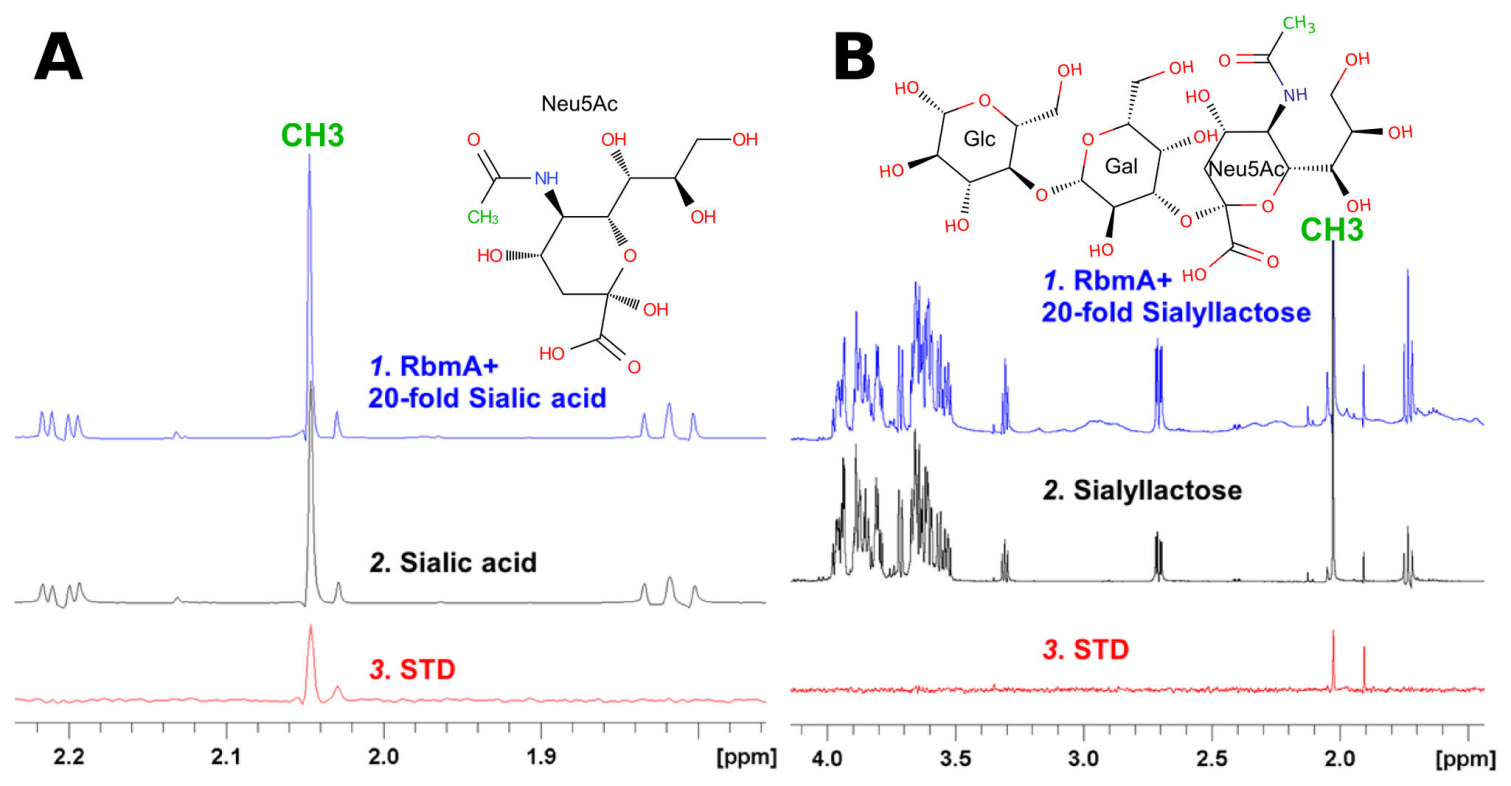
Saturation transfer difference experiments for sialic acid and sialyllactose. A: STD experiments between RbmA and sialic acid. B: STD experiments between RbmA and sialyllactose. The experimental details are described in Methods. The acetyl CH_3_ groups exhibiting saturation transfer difference effect are labeled in green in each figure.

In order to complement our findings on the glycan array, we performed saturation transfer difference (STD) NMR experiments[10]. In these, the saturation (signal attenuation) on a buried methyl group signal propagated throughout the entire protein, and then to the bound-substrate via spin-spin diffusions. Based on the STD effect, it appeared that both sialic acid and sialyllactose (similar to glycan 223) used acetyl methyl groups to bind RbmA, suggesting a very localized binding contact (Figure 3). Further, even though sialyllactose contains α-sialic acid, experiments demonstrate that neither the galactoside, nor the glucoside moieties interact directly with the protein, suggesting that an α-linked negative glycan probably cannot extensively interact with RbmA. Galactose, fucose, and rhamnose STD experiments also demonstrate specific binding to RbmA (Figure S3 in File S1). Galactose and fucose appear to bind mainly via the C3-C4 diol region (Figure S3 A and B in File S1), while the binding of rhamnose is much more extensive, with STD signals for the protons on C2, C4, and C5 (Figure S3 C in File S1).

Galactose and GalNAc are building blocks of VPS, whereas sialic acid, fucose, and rhamnose, for which RbmA exhibited preference, are not[35]. On the other hand, all four non-charged sugars can be found in the O-antigen of different *V. cholerae* strains. Sialic acid is represented on the Vibrion surface by analogues like 3-Deoxy-D-*manno*-oct-2-ulosonic acid, or even N-acylated derivatives of D-perosamine[35–41], hinting at a mechanism for RbmA-cell attachment. *V. cholerae* strains have been mainly characterized as O1, O139 and non-O serotypes. While, within the first two groups, O-antigens are well conserved, the third one is much more diverse. By comparing the first and the third groups [28] and searching for RbmA in their respective genomes, we noticed that many non-O strains did not encode for the *RbmA* gene. Those that did, and some of the O1 serotype strains, presented a series of mutations within the tight groove area, and at the wide groove interface (Figure S4 in File S1). These mutations directly affect the charge and polarity within the O-loop pocket, and of the complementary stripes within the wide groove, thus altering their specificity. Further, it is not uncommon for microbial adhesins involved in biofilm, and surface adhesion to be very plastic, accepting a relatively diverse set of ligands[42–44], and it is therefore not unreasonable to expect RbmA to be able to acommodate related, yet diverse, ligands.

**Figure 4:**
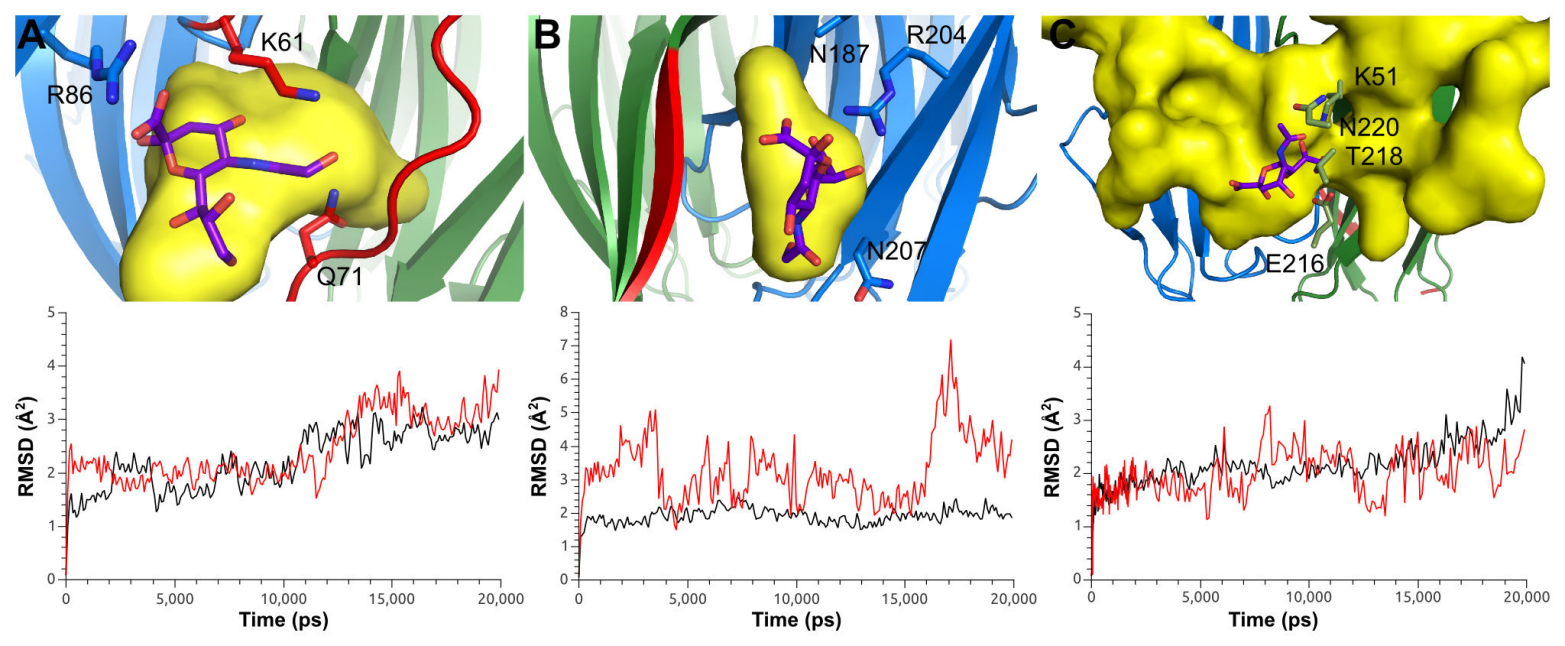
Docking and molecular dynamics simulations of RbmA●sialic acid complexes. Top: RbmA is shown in blue and green, with the O- and D-loop conformations (A and B, respectively) highlighted in red. InCa sitefinder results are shown as yellow surfaces, with the sialic acid docking poses superimposed. Sialic acid is shown in purple, with labeled amino-acids defined as flexible for docking. Bottom: Amber12 md RMSD trajectories for the protein backbone (black) and the glycan (red). A: RbmA●sialic acid within the O-loop pocket. B: RbmA●sialic acid at the D-loop conformation surface. C: RbmA●sialic acid at the wide groove pocket. Other conformations were tested, resulting in less stable binding (Fig. S4 in file S1 and movies S4-S8).

In RbmA, STD experiments suggested that the region surrounding the positive O-loop pocket (Figure S5 in File S1) is the most likely candidate for strong binding with negatively charged ligands, while the D-loop conformation exposes the positive charges in a flat and open region, allowing for a first interaction with the bacterial surface. With two O-loop pockets per functional unit, RbmA would bidentaly recognize two ligands, directly mediating cell-cell contacts. This hypothesis was further tested via extensive *in-silico* screening of the protein surface. As expected, the O-loop pocket and the wide groove were readily identified as good binding regions by the InCa sitefinder server[20], and via docking with sialic acid (Figure 4A, and C, and S2 and S6 in File S1). For the D-loop conformation, several small pockets were detected (Figure 4B, and S2 in File S1). Further confirmation via molecular dynamics indicated that binding was possible for the first two, but not for the D-loop conformation, which was only marginally stable (Figure 4).

**Figure 5:**
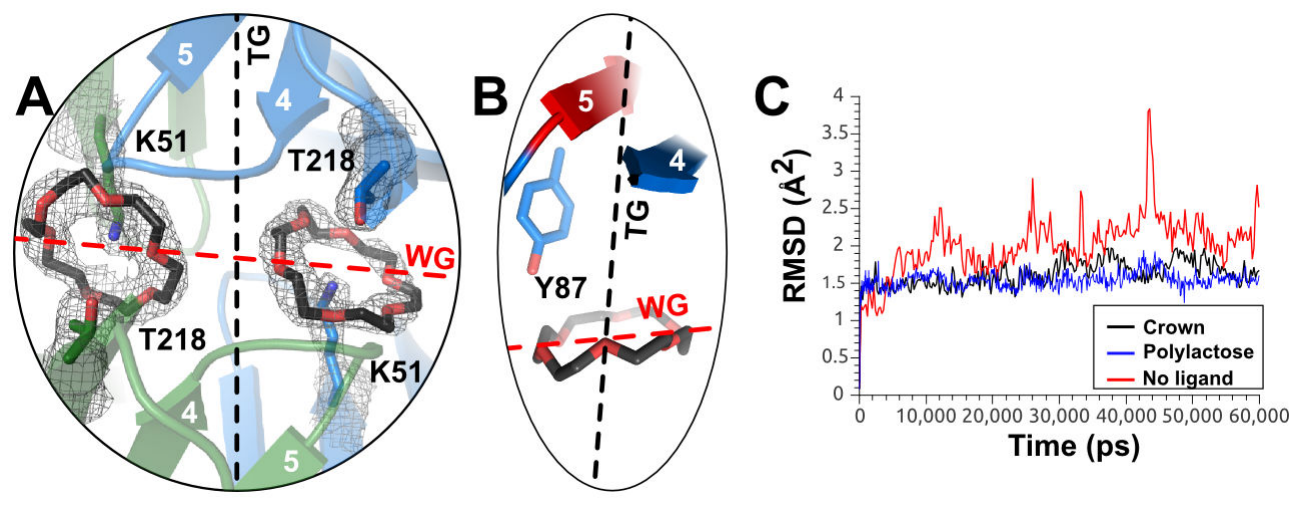
The binding of 18-crown-6 ethers. A: Detail of the RbmA●18-crown-6 binding pocket. One crown ether was bound in the wide groove by each monomer via K51 (A-domain) and T218 (B-domain). B: Detail of the interaction between the 18-crown-6 ether and Y87, which might stabilize β5 in the O-loop conformation. C: RMSD traces of free RbmA (red), forming a complex with experimental crown ethers (black), or a modeled poly-lactose chain (blue, movies S2, S1, and S3, respectively). While, in the presence of the 18-crown-6 ligand, or a polylactose chain, the average position of the RbmA backbone stays constant for 60 ns, while the apo structure is generally less stable, with strong, occasional flickering every ~10 ns.

A thorough co-crystallization screening with various carbohydrates was unsuccessful. Furthermore, soaking experiments with a variety of monosaccharides resulted in the crystals immediately dissolving. As an alternative, a co-crystallization of RbmA with the 18-crown-6 ether was attempted. Crown ethers bind proteins, bridging axially bound lysines to polar amino-acids, with equatorial interactions with tryptophanes and other aromatic residues (Lee, C.-C. et al. in press), and have been shown to occlude ligand accessible channels[45]. Since negatively charged glycans can bind to similar pockets[20], a crown ether was used as a probe in co-crystallization experiments (Figure 5A). These dramatically changed the crystallization behavior of RbmA, and resulted in the emergence of a new crystal form with eight molecules per asymmetric unit (PDBID 4bei). Within each chain, one 18-crown-6 ether molecule was deeply embedded in the wide groove, bound between K51 in the A-domain, and T218 in the B‑domain. Furthermore, all dimers containing crown-ethers had their tight grooves protected by the O-loop conformation, perhaps due to the interaction between the 18-crown-6 ethers and Y87 (Figure 5B).

Crown ether dependent protein stability was studied via 60 ns molecular dynamics simulations (Figure 5C and S7 in File S1). In the absence of the crown ether, strong flickering occurs within the wide groove, indicating a change between an open and a closed conformation. In the presence of the crown ether, flickering is reduced, with the protein closing around the ligand, further confirming the wide groove as a good candidate for ligand binding. A similar behavior could be observed when a poly-lactose oligosaccharide was modeled into the wide groove (Figure 5C and S7 in File S1, and movie S3), suggesting that this structural feature is a suitable binding pocket for polysaccharides. Our results support previous data, which had suggested that RbmA adopts a more extended conformation in solution[33]. Our results, however, additionally demonstrate that the wide groove is capable of accomodating large substrates. These stabilize RbmA in the closed, compact conformation, which is preferentially adopted in the crystal.

## Conclusion

Taken together, our results suggest that RbmA presents two distinct binding sites. On the one hand, the wide groove is capable of accommodating large, filamentous substrates, such as VPS. On the other hand, the O-loop pockets are capable of stable binding of negatively charged carbohydrates, which are a hallmark of cellular surfaces. Our glycan array data, confirmed via STD experiments, indicates that RbmA preferentially binds monosaccharides from the VPS and from the LPS. A diverse pool of ligands is a hallmark of other biofilm-related adhesins[42], and may suggest multivalent binding to several components of the matrix and/or cellular surfaces. Furthermore, the importance of the sialic acid amide group in binding suggests that acylated perosamines, a key component in *V. cholerae* O-antigen[40,46], or acidic glycans would be suitable binders. 

**Figure 6:**
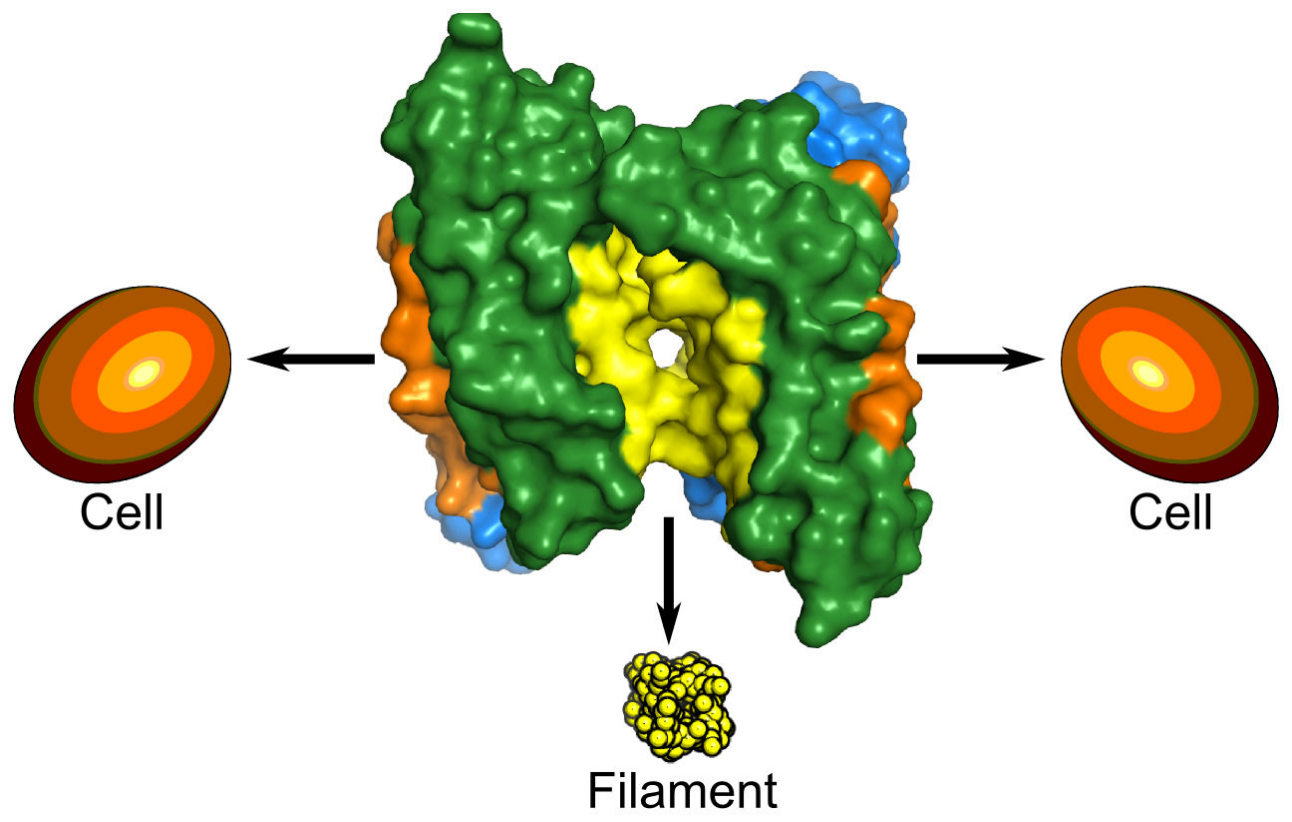
A proposed model for RbmA action. Taking all our results into account, we propose that RbmA (center, green and marine), is capable of binding long, filamentous substrates in its wide groove (yellow surface area) like, for example polysaccharides (bottom, yellow ball model). On the other hand, the positive charges within O-loop pockets on the tight grooves (orange surface areas), might allow RbmA to bind to cellular surfaces (orange ovoid shapes), which are densely negative.

Our structural, and binding studies, suggest a model for biofilm scaffolding (Figure 6), in which RbmA acts as an anchor between cells and between cells and the biofilm, by attaching bidentally to the bacterial carbohydrate-rich surface, but also to a filamentous component of the biofilm, in a similar manner as was proposed by biofilm architectural studies[7]. Giglio et al., with their preliminary studies, showed that the positive charge in the tight groove is important for RbmA function, while they were not able to pinpoint the relevance of the loop conformational transitions [33]. However, our data opens new possibilities for further mutagenesis strategies because we were able to asign possible glycan binding pockets, and established a correlation between O-antigen, and RbmA diversity (Figure S4 in File S1). These, in combination with *V. Cholerae in vivo* studies in its sessile habitat may enhance our understanding on RbmA mediated adhesion and RbmA strain-dependent diversity. Additionally, our characterization of glycan specificity will prove very useful in targeting biofilm inhibition assays. 

## Supporting Information

File S1 **Suppporting Materials.** The supporting materials file contains all supporting figures, and supporting tables.
(PDF)

Movie S1 **Molecular dynamics simulation of the RbmA crown-ether complex.**
(MP4)

Movie S2 **Molecular dynamics simulation of free RbmA.**
(MP4)

Movie S3 **Molecular dynamics simulation of the modeled RbmA -polylactose complex.**
(AVI)

Movie S4 **Molecular dynamics simulation of stable sialic acid-RbmA complex in the O-loop conformation.**
(MP4)

Movie S5 **Molecular dynamics simulation of mildly unstable sialic acid-RbmA complex in the D-loop conformation.**
(MP4)

Movie S6 **Molecular dynamics simulation of stable sialic acid-RbmA complex in the wide groove.**
(MP4)

Movie S7 **Molecular dynamics simulation of unstable sialic acid-RbmA complex in the O-loop conformation.**
(MP4)

Movie S8 **Molecular dynamics simulation of highly unstable sialic acid-RbmA complex in the D-loop conformation.**
(MP4)
