# Supplementary material for: Structural Insights into RbmA, a Biofilm Scaffolding Protein of V. Cholerae
Source: PLoS One. 2013 Dec 5;8(12):e82458. doi: 10.1371/journal.pone.0082458 (PMC3855419; doi:10.1371/journal.pone.0082458)
Supplement: File S1 — Suppporting Materials. The supporting materials file contains all supporting figures, and supporting tables. (PDF) [file pone.0082458.s001.pdf]

Table S1. ΔH values for the sialic acid docking on RbmA (10 best conformations). Conformations employed for md simulations highlighted in bold (Fig. 4 and S3). Those values marked with an asterisk correspond to unstable poses.

	O-loop pocket	D-loop conformation	Wide groove pocket
Pose 1	**-6.6***	**-5.9***	**-7.7**
Pose 2	-6.5	-5.8	-7.6
Pose 3	**-6.3**	-5.7	-7.5
Pose 4	-6.3	-5.6	-7.4
Pose 5	-6.1	**-5.6***	-7.3
Pose 6	-6.1	-5.6	-7.2
Pose 7	-6.1	-5.6	-7.1
Pose 8	-6.0	-5.5	-7.1
Pose 9	-5.9	-5.5	-7.1

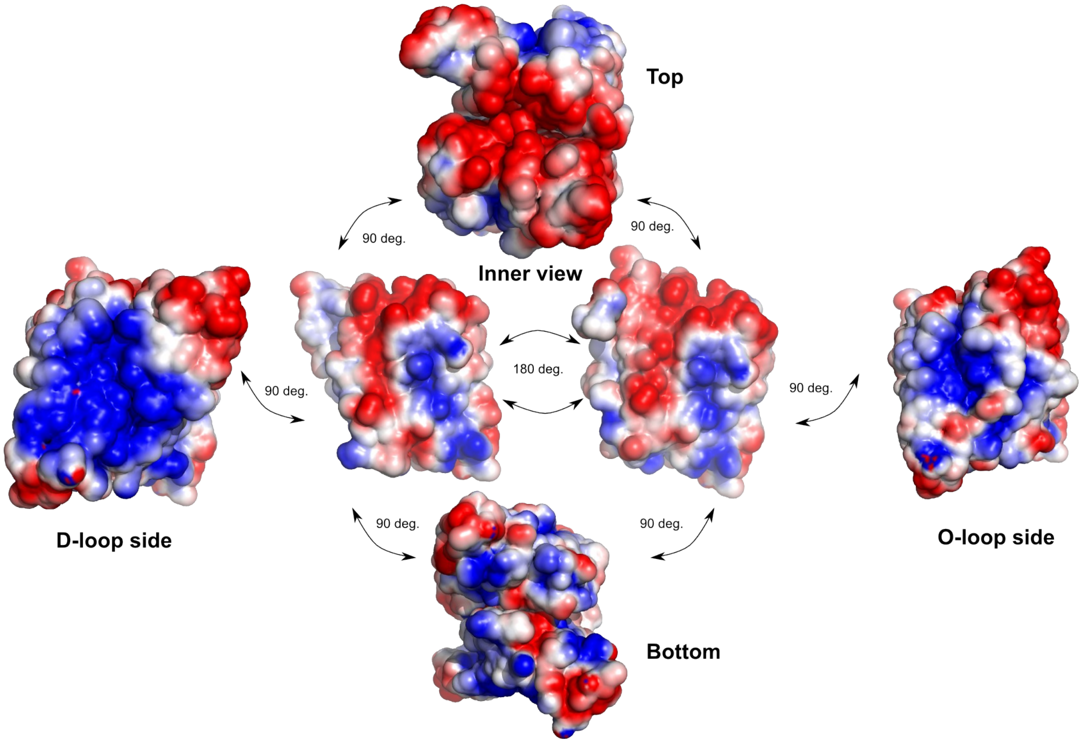

Fig. S1: Apbs electrostatic potentials of RbmA domains along the wide groove. Inner view: The inner sides of RbmA present complementary negatively and positively charged stripes, resulting in attractive forces. Top and Bottom views: On the edges of the interaction, however, positive (bottom) and negative charges (top) are packed closely together, resulting in repulsive forces. On the outer sides (D- and O-loop sides), a large positive patch can be found, which is completely exposed in the D-loop conformation, but partially shielded in the O-loop conformation. All potentials were calculated using apbs, and the apbs plugin for pymol. Electrostatic scale goes from -2 (red) to +2 (blue).

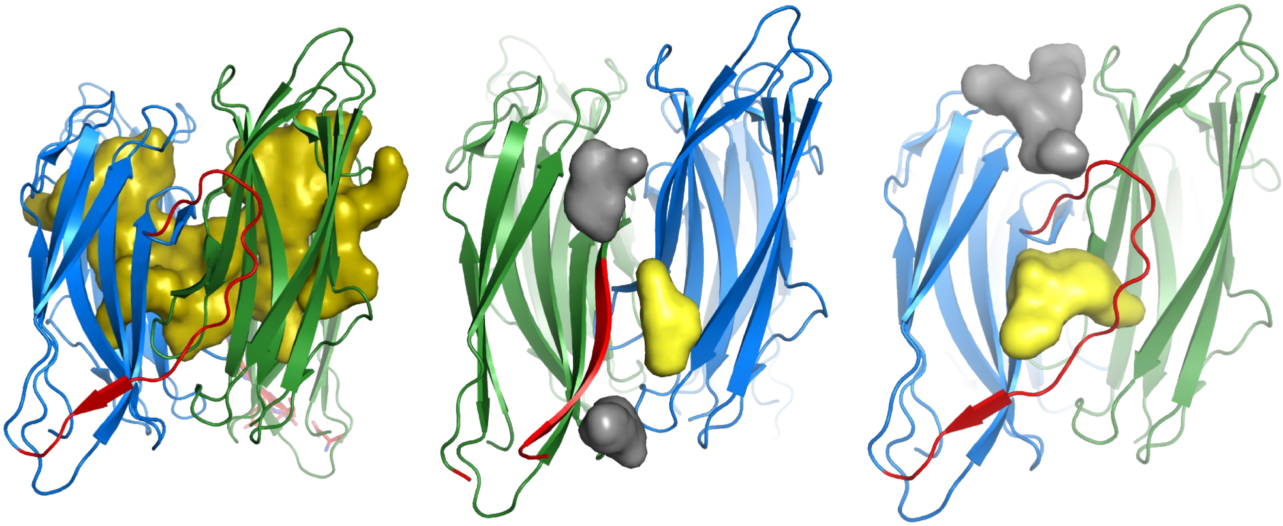

Fig. S2: InCa sitefinder predicted binding sites for RbmA. Binding pockets further corroborated by Vina docking are highlighted in yellow. Left: large binding pocket within the wide groove. Center: binding pockets found in the D-loop conformation. Right: Binding pockets found in the O-loop conformation.

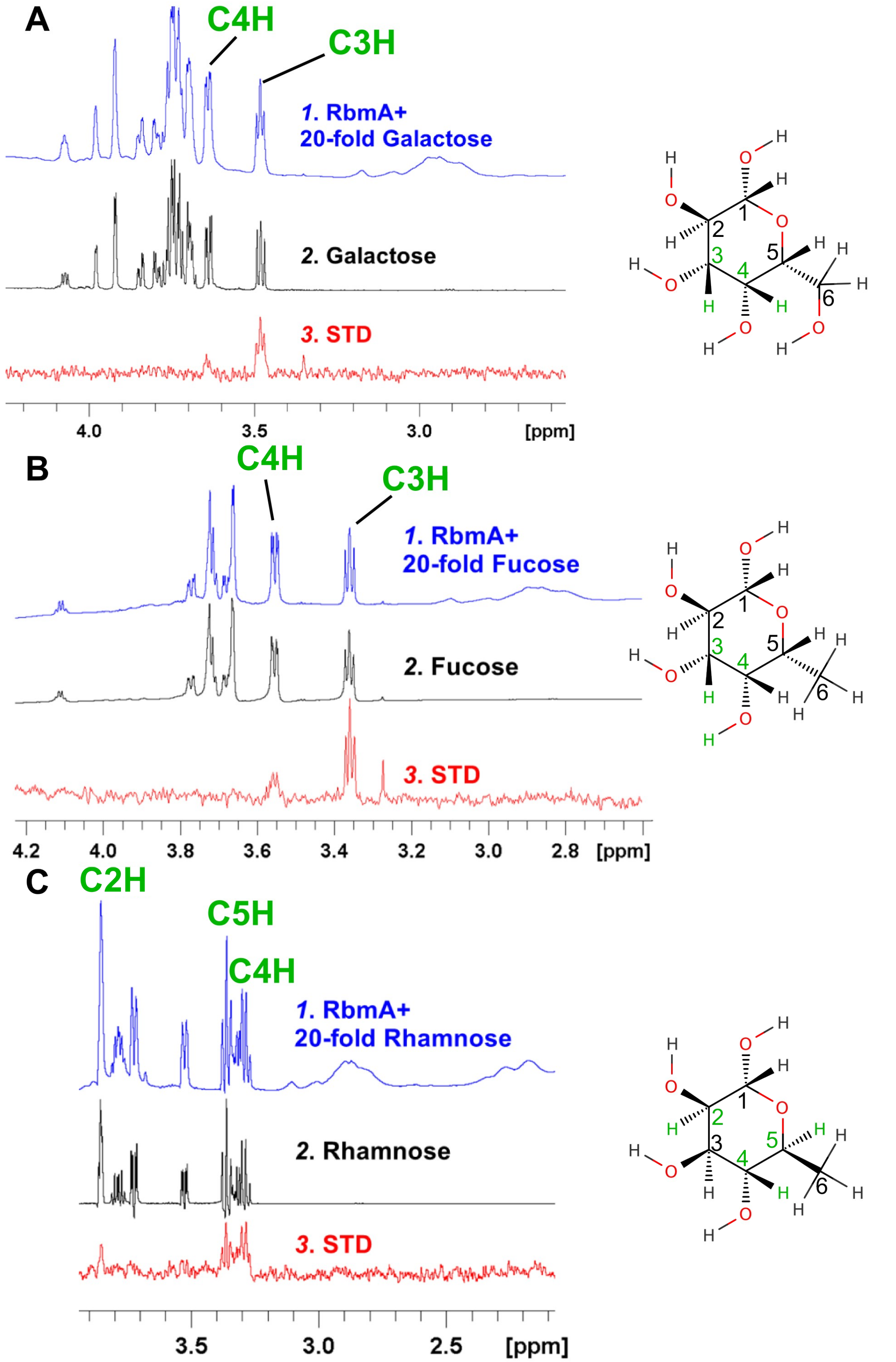

Fig. S3: STD effects for other sugars. Galactose (A), fucose (B) and rhamnose (C) STD spectra are shown here, with assignment for the interacting peaks highlighted in green.

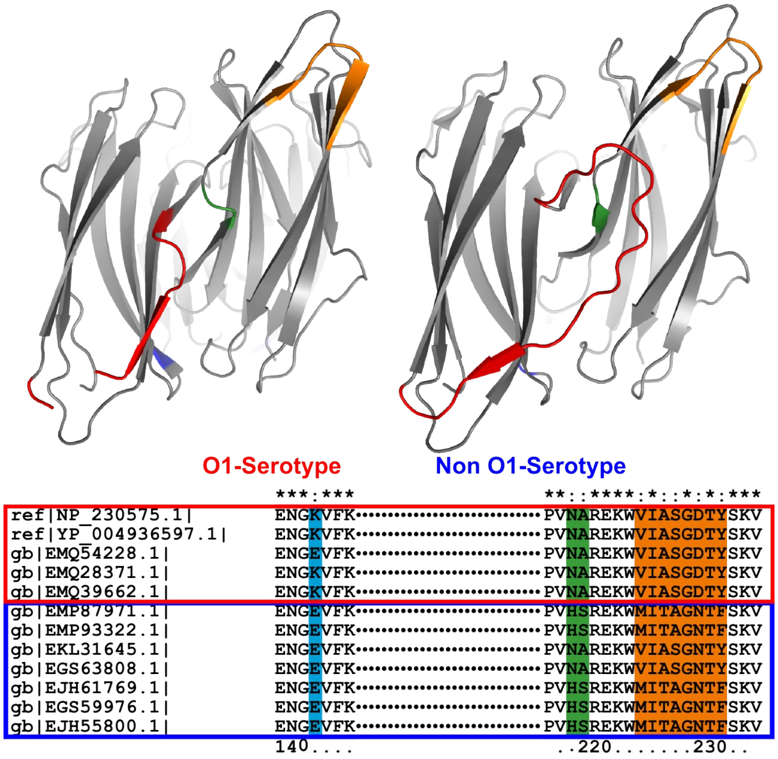

Fig. S4: RbmA multiple alignment and variability centers in different Vibrio cholerae strains. Colors on the structures correspond to the sequences in the alignment. Further, the red highlights in the structure correspond to the D-loop (left) and O-loop (right) conformations. The alignment classification was based on the serotyping performed in [2]

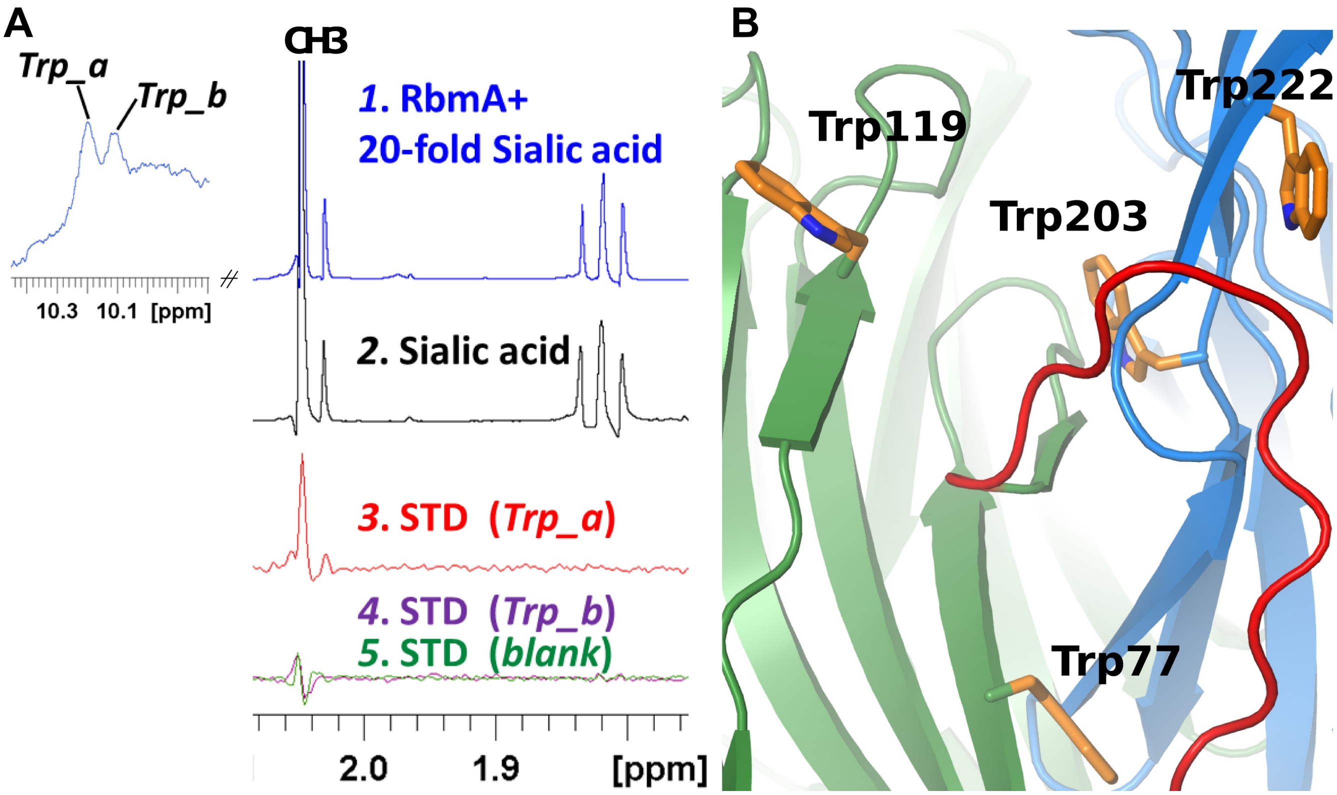

Fig. S5: Differential STD measurements for two tryptophan peaks. A: In the ^1H NMR spectrum of RbmA, two distinct Trp indole N-H peaks were detected, Trp_a, and Trp_b. These two correspond to two different tryptophan residues. B: When STD experiments were performed against sialic acid, at the Trp_a frequency, a clear methyl STD effect was detected. On the other hand, when the sample was irradiated at the Trp_b frequency, there was no detectable STD signal. This suggests that, while Trp_a interacts with sialic acid, Trp_b does not. C: all tryptophans in RbmA are within the tight groove.

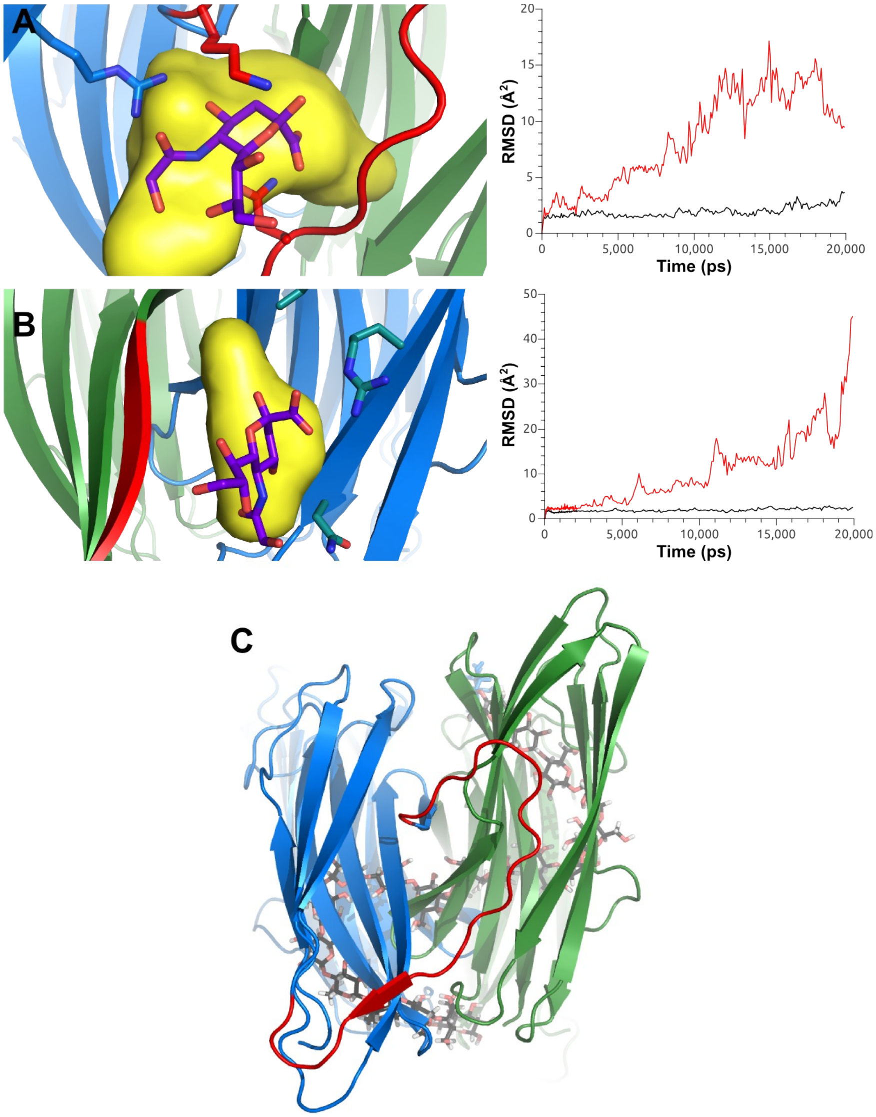

Fig. S6: Modeled sialic acid (unstable) and polylactose poses. InCa sitefinder predicted sites are highlighted in yellow, while sialic acid is presented in purple. On the right, the black curve represents protein backbone RMSDs, while the red curve corresponds to the sialic acid. A: sialic acid pose for the O-loop pocket. B: unstable pose for the D-loop surface. Under these conditions, the RbmA●sialic acid compex was not stable. A corresponds to pose 1 at the O-loop pocket, while B corresponds to pose 5 of the D-loop conformation. C: RbmA with the modeled polylactose.

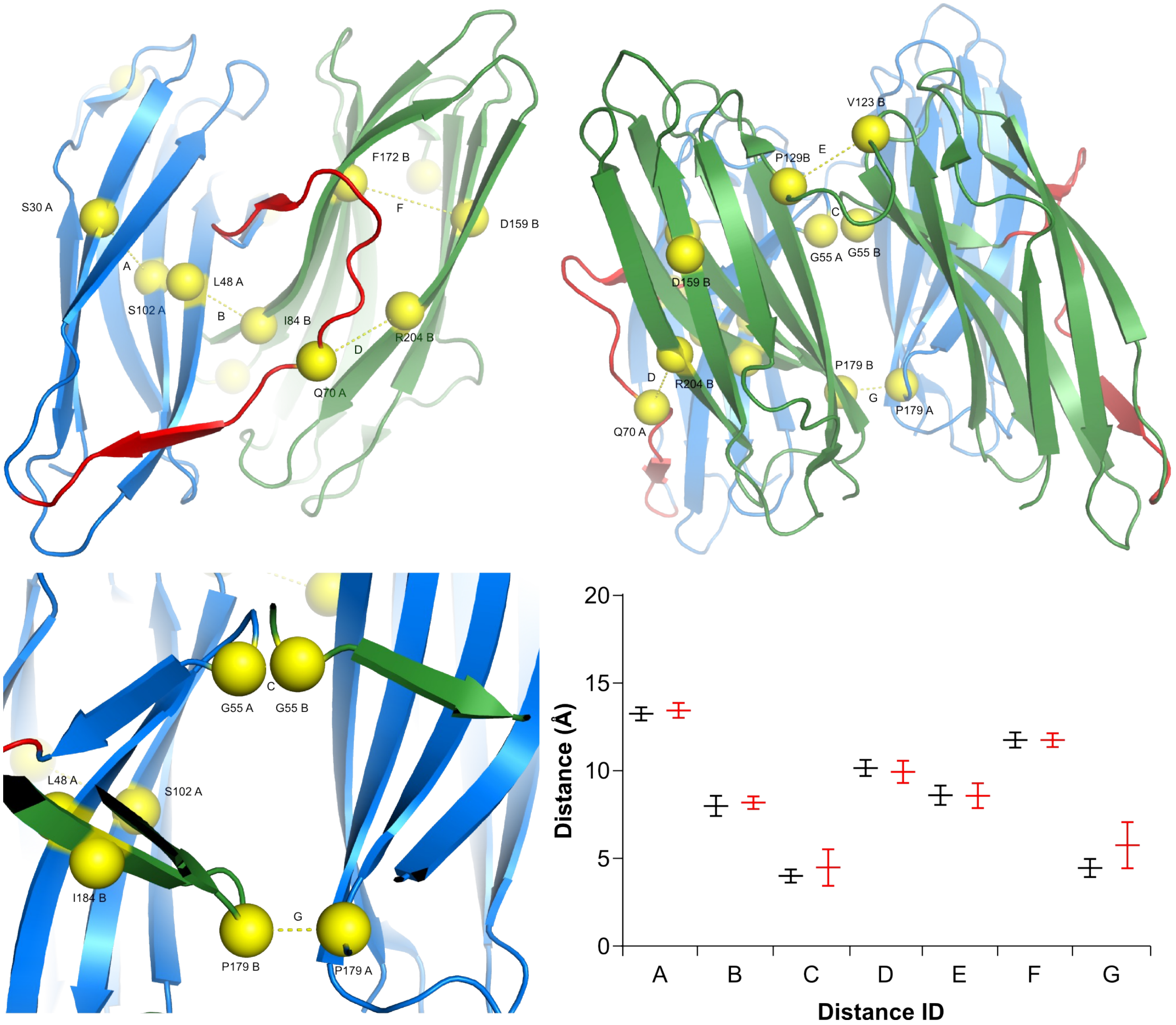

Fig. S7: Detailed description of vibration within RbmA in the presence and absence of crown ether. Cα from selected amino-acids are presented as yellow spheres, while distances between atom pairs are represented by yellow dashed lines and identified by a letter. Top Left: RbmA view along the tight groove. Top right: RbmA view along the wide groove. Bottom left: detail within the wide groove, highlighting distances C and G, which are the ones within the wide groove. Bottom right: Bar diagram showing average distance and standard deviations for each atom pair in the presence (black) and absence (red) of crown ether. Atom pairs are identified by their distance IDs. A: S30 A chain to S102 A chain monitors movement within the A domain. B: L48 A chain to I184 B chain monitors movement along the tight groove. C: G55 A chain to G55 B chain monitors movement within the wide groove. D: Q70 A chain to R204 B chain monitors movement at the O-loop A/B interface. E: P129 B chain to V123 B chain monitors distance changes along the hinge loop. F: D159 B chain to F172 B chain monitors movement within the B domain. Distance changes between atom pairs A, B, E, D, and F remain fairly constant with or without the crown ether ligand. On the other hand, atom pairs C and G, which are the ones present within the wide groove, show a marked increase in both average distance and standard deviation. Therefore, the increased flickering along the wide groove in the absence of crown ether contributes to the overall RMSD increase presented in Fig. 1E.

1. Linke D, Goldman A (2011) Bacterial Adhesion: Chemistry, Biology and Physics. New York: Springer 299 p.

2. Hasan NA, Choi SY, Eppinger M, Clark PW, Chen A, et al. (2012) Genomic diversity of 2010 Haitian cholera outbreak strains. P Natl Acad Sci Usa 109: E2010–7.
